# Burnout syndrome among medical students in Kazakhstan

**DOI:** 10.1186/s40359-022-00901-w

**Published:** 2022-08-06

**Authors:** Aidos K. Bolatov, Telman Z. Seisembekov, Dariga S. Smailova, Hengameh Hosseini

**Affiliations:** 1 NpJSC, Astana Medical University, Beybitshilik St. 49A, Z10K9D9 Nur-Sultan, Kazakhstan; 2 University Medical Center, Kerey-Zhanibek Handar St. 5/1, Z05P3Y4 Nur-Sultan, Kazakhstan; 3 Kazakhstan School of Public Health, Utepov St. 19A, A15T6B7 Almaty, Kazakhstan; 4 University of Scranton, 800 Linden St., Scranton, PA 18510-4699 USA

**Keywords:** Burnout, Medical students, Kazakhstan, Copenhagen Burnout Inventory, Oldenburg Burnout Inventory

## Abstract

**Background:**

Burnout is a serious problem in the training and professional development of medical students. However, there is no known data on the prevalence of burnout among medical students in Kazakhstan. This study aims at investigating burnout and associated factors in a sample of students from Astana Medical University.

**Methods:**

The study included socio-demographic and personal questions, Oldenburg Burnout Inventory for college students (OLBI-S) and Copenhagen Burnout Inventory-Students survey (CBI-S) to measure burnout. Statistical analyses included measures of descriptive statistics and regression analysis for evaluating burnout-associated factors.

**Results:**

In total, 736 medical students responded. The prevalence of burnout syndrome was 28% (CBI-S) and 31% (OLBI-S). There was a significant association between the prevalence and the level of burnout and student’s gender, year of study, thoughts of dropping out, suicidal ideation, satisfaction with the chosen profession and academic performance, interpersonal relationship problems, the decision to study in medical school, smoking, accommodation, parental expectations, alcohol use, extracurricular activities, part-time job, somatic symptoms, depression, and anxiety.

**Conclusions:**

The factors associated with burnout were identified, which complements and expands the existing data on academic burnout. The data obtained can help in organizing psychological assistance for medical students in Kazakhstan.

## Introduction

In recent years, the mental health of medical students and residents has been a growing concern as it has become increasingly clear that burnout is serious problem, which led to the introduction of institutional initiatives to combat this phenomenon [1, 2]. Medical students are constantly exposed to psychosocial stressors throughout their studies, which, if persistent, can lead to burnout [3]. Various predictors of academic burnout have been previously studied. But not always certain factors had the same and/or significant impact on the level of burnout and possibly depended on the culture, education system, country and period of study. Thus, in a systematic review, Frajerman et al. [4] found no significant association between gender and burnout. In contrast, two systematic reviews of Chinese and Brazilian medical schools found higher levels of burnout in males [5, 6]. Some prior research has found that burnout was not associated with different types of extracurricular activities [7, 8]. However, it is known that extracurricular activities like those involving music and physical exercise may reduce burnout and other mental health problems [9, 10]. Therefore, it is important to understand that the different factors that students encounter during their studies and those that surround a person throughout life may affect their mental well-being in different ways. For instance, the degree to which students feel to have been empowered to make their own decisions regarding studies and career seems to influence susceptibility to burnout. We note that the culture of Kazakhstan is distinct from that of more individualistic nations’ cultures that heavily promote independence and autonomy of young adults in that it is common for younger generations to strictly obey and yield to the opinions of older relatives. This influence often extends to decisions about which specialty children should pursue at university.

Academic burnout is more common among medical students and affects their mental health, academic performance and interpersonal relationships [11, 12]. Increasing stress associated with lengthy learning processes and the academic environment, which presents students with many challenges [13]. Such consequences of emotional burnout can be considered depression and poor psychosomatic state [14, 15]. Studies conducted among US medical students by Dyrbye et al. [16] and Dyrbye and Shanafelt [17] found that burnout can contribute to suicidal ideation, while recovery from burnout decreases the prevalence of suicidal ideation. Data from the systematic review of Ishak et al. [18] pointed to an association between burnout in medical students and suicidal ideation. Particular attention should be paid to the fact that Kazakhstan has one of the world's highest overall suicide rates [19]. Moreover, data collected from three medical universities in Kazakhstan indicate that the prevalence of suicidal ideation among first-year students was 8.9% [20]. According to the Institute for Health Metrics and Evaluation (University of Washington School of Medicine) in Kazakhstan, the death rate from substance use among people aged 15–49 increased by 63.6% from 1990 to 2019, and ranks 7th among the causes of death in this age group [21]. Substance use seems to play a meaningful role in burnout. Results obtained by Cecil, et al. [22] indicated that being an ex-smoker was significantly predictive of higher emotional exhaustion scores. Also, a study from Japan concluded that the mental health status of dental students among regular smokers was better than that of non-current smokers [23]. One longitudinal survey among German and Chinese students showed healthy lifestyle choices like choosing not to smoke are related to improvements in mental health over 1 year [24]. On the other hand, a study from South-West Ethiopia indicates that smoking cigarettes was significantly associated with common mental disorders [25]. Alcohol use was also of note in the burnout research. Jackson et al. [26] found that alcohol abuse or dependence was more common among medical students with burnout, high emotional exhaustion, and high levels of depersonalization. Research among British medical students did not find significant correlations between any of the personality variables and alcohol consumption [27]. Cecil et al. [22] reported that increased alcohol binge scores were significantly associated with higher personal accomplishment scores.

Considering the importance of understanding the issues of academic burnout in the professional development of medical students, as well as the risks of deterioration in prevalence and the consequences of burnout, there are few data on the burnout of medical students in Kazakhstan. Several previous studies in Kazakhstan concluded a high level of "reduction of personal achievements" among 2 years students and a high rate of "depersonalization" among 5 years students [28], at once emotional exhaustion among students was moderately expressed [29]. Moreover, in the system of Kazakhstani medical education, insufficient attention is paid to this problem. Thus, current study aims at investigating burnout prevalence and related factors in a sample of students at Astana Medical University (Nur-Sultan, Kazakhstan). To the best on our knowledge, this is the first complex study on burnout syndrome among medical students in Kazakhstan.

There are several methods for assessing burnout, however, according to a systematic review conducted by Shoman et al. [30], CBI and, in to a lesser extent, OLBI showed robust psychometric properties among burnout measures. Moreover, current scales have a different factorial structure (OLBI includes two dimensions, exhaustion and disengagement; CBI focuses only on fatigue/emotional exhaustion, but measures in the different life domains [31, 32]). Therefore, it was decided to use both scales to create a broader understanding of the structure of the burnout phenomenon.

## Materials and methods

### Study design

This cross-sectional questionnaire-based study was carried out during the period October–December, 2019.

### Procedure

This study was conducted as part of an initiative project to study the psychological well-being of medical students in Kazakhstan. In the beginning, adaptation and validation of scales for assessing burnout were carried out [33, 34], after which, in this study, we evaluate burnout and associated factors. Medical students at Astana Medical University were asked to anonymously complete an online survey created on the 1 ka platform (www.1ka.si).

### Measurement

The questionnaire included:

- Items on socio-demographic and personal characteristics (sex, age, year in medical school, accommodation, part-time job, extracurricular activities, suicidal ideation, thoughts of dropping out, relationship problem with family and friends, satisfaction with academic performance and chosen profession, smoking and alcohol use, and pursuit of high parental expectations).
- Burnout syndrome was assessed using the Oldenburg Burnout Inventory for college students (OLBI-S) [31] and the Copenhagen Burnout Inventory (CBI) [32], adopted for students by Campos et al. [35]—CBI-S, translated and validated by authors [33, 34].
- The CBI-S consists of 25 items that represent four dimensions: Personal Burnout, Studies-Related Burnout, Colleague-Related Burnout, and Teacher-Related Burnout (TRB).The answers that can be given to each item were “always = 100”, “frequently = 75” “sometimes = 50” “rarely = 25” and “never = 0”, with inverse scoring for item 10. For each scale, a total average score was calculated. A burnout level (severity) was assessed according to Kristensen’s criteria [36]. Internal consistency of CBI-S among current sample was excellent (Cronbach’s α = 0.942) [37].
- The OLBI-S includes 16 items defined in 2 subscales: Exhaustion and Disengagement. Each subscale includes 8 items that are scored on a 4-point Likert scale from 1 “strongly agree” to 4 “strongly disagree”. Burnout criteria were taken as those of Peterson et al. [38]. In current research internal consistency of OLBI-S was reliable (Cronbach’s α = 0.889) [37].
- Common physical symptoms were assessed by the Patient Health Questionnaire-15 (PHQ-15) scale. It consists of 15 items, with a score of 0–2 points for each item, and 30 points total. A cutoff score of 5, 10, and 15 points indicates low, medium, and high severity of somatic symptoms, respectively [39]. Students scoring higher than 10 were considered having severe somatic symptoms [40]. PHQ-15 has demonstrated reliable internal consistency (Cronbach’s ɑ = 0.824).
- The Generalized Anxiety Disorder, the 7-item (GAD-7) scale was used to assess anxiety over the past 2 weeks [41]. Each question had four possible answers and ratings: “Not at all” (0), “Several days” (1), “More than half the day” (2), “Nearly every day” (3). The total score was calculated according to the results and interpreted as follows: 0–4 scores (Minimal), 5–9 scores (Mild), 10–14 scores (Moderate), and 15–21 scores (Severe). Participants with scores higher than 10 were considered to be anxious [40]. GAD-7 has demonstrated strong internal consistency (Cronbach’s ɑ = 0.925).
- Depression was used using the 9-item Patient Health Questionnaire-9 (PHQ-9) [42]. The participant had to respond to the question: “How often have they been bothered by the following (by what) over the past 2 weeks?” Each question had four possible answers and ratings: “Not at all” (0), “Several days” (1), “More than half of the days” (2), “Nearly every day” (3). The total score was calculated and interpreted as follows: 0–4 scores (Minimal or none), 5–9 scores (Mild), 10–14 scores (Moderate), 15–19 scores (Moderately severe), and 20–27 (Severe). Participants with scores higher than 10 were considered depressed [40]. PHQ-9 has demonstrated reliable internal consistency (Cronbach’s ɑ = 0.899).
- Suicidal ideation was assessed by asking students: “Have you ever had thoughts of taking your own life while you were in medical school?” We assessed thoughts of dropping out by asking students if they had had any thoughts of dropping out of medical school in the past 12 months. Students who responded “yes” to these two questions were rated as having suicidal ideation and thoughts of dropping out, respectively.
- Substance use was assessed using the question "Do you drink alcohol / do you smoke?" Those students who answered yes to these questions were additionally asked the question "When did you start drinking/smoking?" to which there were two possible answers "Before entering medical school" or "After entering medical school". Those respondents who started drinking/smoking before entering medical school were also asked, "If you started drinking/smoking before entering medical school, has your drinking/smoking increased or decreased since entering to medical school?"

### Participant characteristics

In total, 1209 students were survived, and among them 736 students completed the questionnaire (response rate = 60.9%). Moreover, the final sample exceeds the minimum size equal to 356 (given a population size of 4931 students in a given academic year (2019–2020), confidence level 95%, and confidence level 5%).The mean age of the respondent was 20.3 years (17–33, SD = 2.74). Table 1 presents baseline socio-demographic and personal data of participants. Three-quarters of the participants were female and undergraduate students (1–5 years students).

**Table 1:** Characteristics of the study population. Rate ratio (RR) of factors associated with burnout among medical students (N = 736)

Characteristics (n, %)	Burnout (CBI-S) n (%)	RR	Burnout (OLBI-S) n (%)	RR
*Gender (n = 736)*				
Female (552, 75.0)	153 (27.7)	1	161 (29.2)	1
Male (184, 25.0)	53 (28.0)	1.04	67 (36.4)	1.25
*Year in medical school (n = 736)*				
1st year (202, 27.4)	40 (19.8)	1	33 (16.3)	1
2nd year (130, 17.7)	52 (40.0)	2.02**	54 (41.5)	2.54**
3rd year (102, 13.9)	31 (25.5)	1.53	41 (40.2)	2.46**
4th year (55, 7.5)	14 (29.1)	1.47	16 (29.1)	1.78*
5th year (66, 9.0)	18 (27.3)	1.38	13 (19.7)	1.21
Interns (142,19.3)	39 (27.5)	1.39	58 (40.8)	2.50**
Residents (39, 5.3)	10 (25.6)	1.30	13 (33.3)	2.04*
*Decision to study at a medical school (n = 736)*				
Its own decision (573, 77.9)	139 (24.3)	1	148 (25.8)	1
Parents' decision (112, 15.2)	46 (41.1)	1.69*	51 (45.5)	1.76**
Other reason (51, 6.9)	21 (41.2)	1.70*	29 (56.9)	2.20**
*Satisfaction with the chosen profession (n = 538)*^*#*^				
No (190, 35.3)	94 (49.5)	2.36**	121 (63.7)	3.21**
Yes (348, 64.7)	73 (21.0)	1	69 (19.8)	1
*Satisfaction with academic performance (n = 541)*^*#*^				
No (251, 46.4)	109 (43.4)	2.10**	113 (45.0)	1.65**
Yes (290, 53.6)	60 (20.7)	1	79 (27.2)	1
*Thoughts of dropping out (n = 539)*^*#*^				
No (338, 62.7)	57 (16.9)	1	58 (17.2)	1
Yes (201, 37.3)	111 (55.2)	3.28**	134 (66.7)	3.89**
*Accommodation (n = 736)*				
In student dormitory (132, 17.9)	28 (21.2)	1	29 (22.0)	1
Rental housing (198, 26.9)	56 (28.3)	1.33	66 (33.3)	1.52*
At home (406, 55.2)	122 (30.0)	1.42	133 (32.8)	1.49*
*Part-time job (n = 736)*				
No (549, 74.6)	149 (27.1)	1	162 (29.5)	1
Yes (187, 25.4)	57 (30.5)	1.12	66 (35.3)	1.20
*Extracurricular activities (n = 736)*				
No (462, 62.8)	119 (25.8)	1.23	153 (33.1)	1.21
Yes (274, 37.2)	87 (31.8)	1	75 (27.4)	1
*Pursuit of high parental expectations (n = 537)*^*#*^				
No (229, 42.6)	60 (26.2)	1	59 (25.8)	1
Yes (308, 57.4)	109 (35.4)	1.35	133 (43.2)	1.68**
*Interpersonal relationship problem (n = 541)*^*#*^				
No (409, 75.6)	103 (25.2)	1	126 (30.8)	1
Yes (132, 24.4)	66 (50.0)	1.99**	66 (50.0)	1.62**
*Suicidal ideation (n = 539)*^*#*^				
No (456, 84.6)	108 (23.7)	1	133 (29.2)	1
Yes (83, 15.4)	59 (71.1)	3.00**	57 (68.71)	2.36**
*Smoking (n = 548)*^*#*^				
No (474, 86.5)	137 (28.9)	1	151 (31.9)	1
Yes (74, 13.5)	34 (45.9)	1.59*	42 (56.8)	3.31**
*Alcohol use (n = 547)*^*#*^				
No (420, 76.8)	122 (29.0)	1	122 (29.0)	1
Yes (127, 23.2)	49 (38.6)	1.34	71 (55.9)	1.93**
Total (N = 736)	206 (28.0)		228 (31.0)	

*CBI-S* Copenhagen Burnout Inventory-Students survey, *OLBI-S* Oldenburg Burnout Inventory for college students

*p < 0.05, **p < 0.001

^#^Missing responses were excluded from the total before percentages and OR was calculated

### Statistical analysis

Data analysis was conducted using SPSS version 20.0 and Jamovi version 1.2.17.

Descriptive statistics were performed using mean and confidence intervals (95% CI) for quantitative variables. Percentages were computed for qualitative variables. These data analysis methods were used to describe the socio-demographic characteristics of the study population. Independent sample *t* test and ANOVA with post-hoc test were used to assess the differences of variables with a normal distribution between two and more than two groups, respectively. We performed χ2-test, correlation, logistic and linear regressions to evaluate independent associations of the independent variables with burnout. A statistically significant difference was accepted at a p-value of less than 5%.

### Ethics approval

The study was approved by the Local Ethics Committee of the NpJSC “Astana Medical University” (extract from protocol No. 3, held on September 20, 2018).

## Results

In total, 736 students took part in the study, among whom 552 (75%) females and 184 (25%) males. Initially, an overall assessment of the prevalence of burnout among a sample of medical students was carried out. CBI-S mean score was 39.8, and mean subscale scores for this sample were PB (52.5 ± 21.6), SRB (50.5 ± 23.4), CRB (23.6 ± 21.2), and TRB (32.7 ± 24.2). The distribution of the burnout syndrome severity by total CBI-S scoring was as follows: no\low—72.0%, moderate—25.3%, high—2.6%, and severe—0.1%. OLBI-S mean score for Exhaustion subscale was 2.79 (SD = 0.628), for Disengagement—2.43 (SD = 0.631). Distributions of OLBI-S dimensions by severity were low (23.9%), average (59.9%) and high (16.2%) for Exhaustion, and low (17.4%), average (59.1%) and high (23.5%) for Disengagement.

Next, we studied the comparative indicators of the level and prevalence of burnout in various socio-demographic groups of students. For this, methods of comparative analysis of average values and regression analysis were used. Thus, there was no significant difference in the prevalence of burnout by gender according both CBI-S and OLBI-S. However, female students demonstrated higher level (53.8 ± 21.2 vs 48.5 ± 22.2) and prevalence (61.2% vs 51.6%) of PB compare to males, p < 0.05. But male students showed higher prevalence of high disengagement (20.3 vs 33.2, p < 0.001) compare to females (Table 2) with RR = 1.63 (95% CI 1.20–2.23, p < 0.05).

**Table 2:** Prevalence of burnout by dimensions (N = 736)

Variables	CBI-S (%)				OLBI-S (%)	
	PB	SRB	CRB	TRB	High exhaustion	High disengagement
*Gender*						
Male	51.6	54.3	16.3	29.9	14.7	33.2
Female	61.2*	54.9	12.0	23.9	16.7	20.3**
*Satisfaction with the chosen profession*						
Yes	48.6	42.0	10.3	20.4	11.2	12.6
No	83.2**	83.2**	17.9*	41.1**	31.6**	54.2**
*Satisfaction with academic performance*						
Yes	50.0	45.9	9.0	20.0	13.1	21.0
No	73.7**	69.3**	17.9*	36.7**	24.7**	35.1**
*Thoughts of dropping out*						
Yes	85.6	86.1	18.4	45.8	31.8	56.7
No	46.4**	39.3**	9.5*	17.2**	10.7**	10.4**
*Part-time job*						
Yes	67.9	60.4	12.3	29.4	13.4	32.1
No	55.7*	52.8	13.3	24.0	17.1	20.6**
*Extracurricular activities*						
Yes	54.4	52.2	17.5	27.7	15.7	18.6
No	61.5*	56.3	10.4*	24.0	16.5	26.4*
Pursuit of high parental expectations (n = 537)*						
Yes	68.2	64.9	14,6	32.5	23.4	32.5
No	52.0**	45.9**	11.4	21.8*	12.2**	21.4*
*Interpersonal relationship problem*						
Yes	82.6	70.5	22.0	42.4	24.2	38.6
No	54.0**	52.3**	10.3**	23.0**	16.6*	24.0**
*Suicidal ideation*						
Yes	88.8	84.3	24.1	54.2	42.2	55.4
No	55.9**	51.5**	11.0**	22.8*	14.3**	22.1**
*Smoking*						
Yes	75.7	78.4	17.6	43.2	27.0	47.3
No	58.4*	53.4**	12.4	25.3**	16.9*	24.3**
*Alcohol use*						
Yes	69.3	66.1	18.1	37.0	26.0	44.9
No	58.3*	54.0*	11.4*	25.0*	16.0*	22.1**
*Total*						
58.8	54.8	13.0	25.4	16.2	23.5	

Cut-off values for PB, SRB, CRB and TRB were > 50, high exhaustion — > 3.25, and > 2.75 for high disengagement

The results are derived from the chi-square test

*PB* personal burnout, *SRB* studies-related burnout, *CRB* colleague-related burnout, *TRB* teacher-related burnout

*p < 0.05, **p < 0.001

**Fig. 1:**
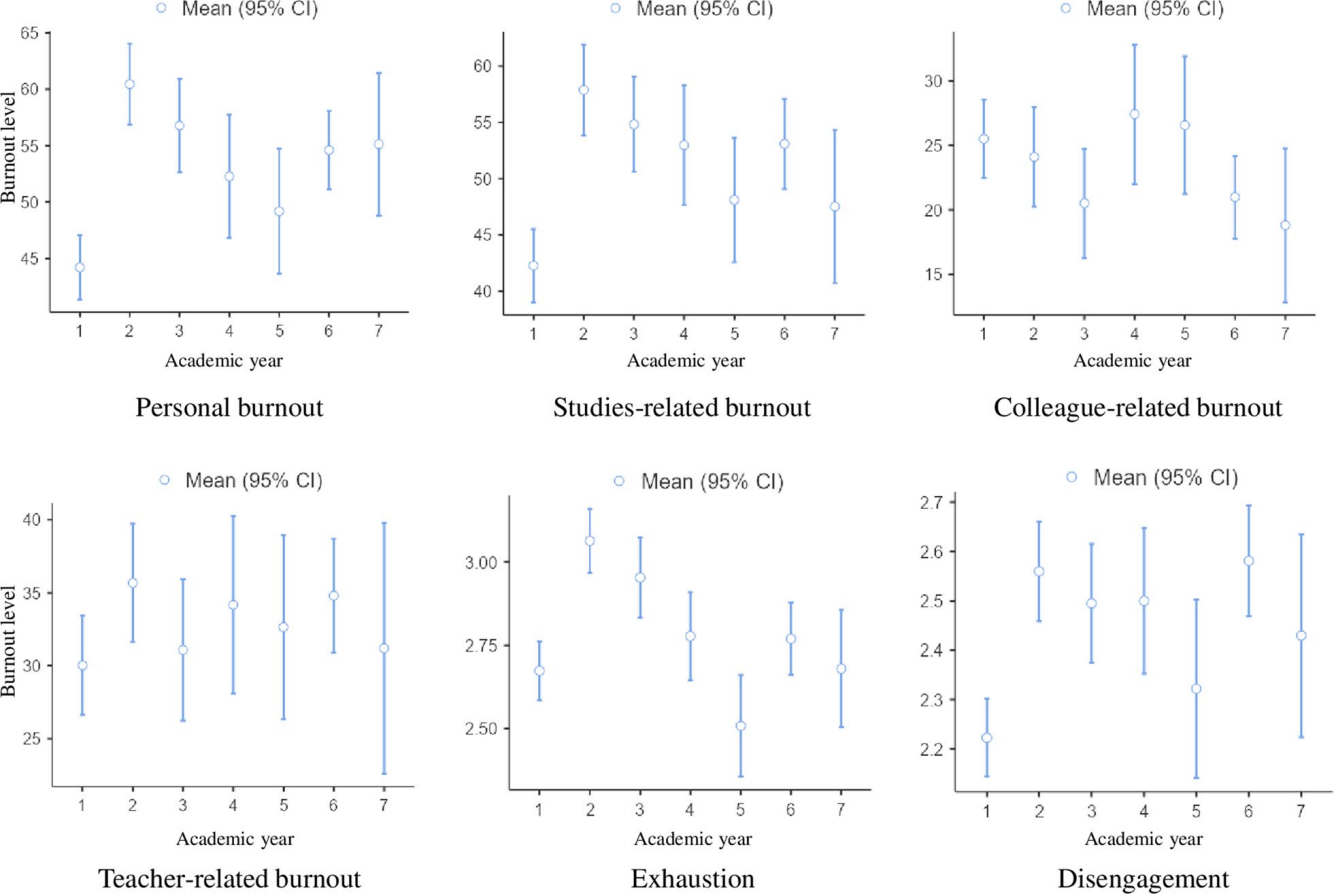
Mean values of burnout dimensions depending on the student’s year of study. *Note* 1–5 correspond to 1–5 years of study, 6—intern students, 7—residents

Based on the CBI-S burnout criterion, the highest incidence was observed among 2-years students (40%). Whereas, according to the criteria of the OLBI-S, the most widespread burnout was found among 2 years (41.5%), 3 years (40.2%), internship students (40.8%), and residents (33.3%). Mean values for burnout sub-scales depending on the year of study presented in Fig. 1. Regression analysis showed that compared with the first year, students in other years of study have 1.4–1.9 times more PB, SRB (p < 0.05). 2-years and 3 years students compare to 1 year 2.0–2.6 times more highly exhausted. 2–5 years undergraduate students, interns and residents compare to 1 year were 1.8–2.6 times more disengaged (p < 0.05).

Dormitory students demonstrate lower personal (48.28 vs 54.38, p < 0.05), studies-related (45.23 vs 52.82, p < 0.05) and total burnout (36.31 vs 41.04, p < 0.05) than students living at home. Moreover, students living in a dormitory had a lower level of studies-related burnout compared to students who occupy non-student housing (45.23 vs 50.91, p < 0.05). Burnout according to OLBI-S criteria was observed less often among students living in a dormitory than among those who lived at home (RR = 0.67, 95% CI 0.45–1.00), p = 0.05.

573 (77.9%) respondents indicated that they chose a medical university by their own decision, while 112 (15.2%) respondents indicated that the decision was made by their parents, and 51 (6.9%) chose other reasons for entering the university. Among students who decided to enter medical school themselves, the incidence of burnout was lower compared to students who indicated parental decision and other reasons, p < 0.001 (Table 1). This is also confirmed by regression analysis: when using CBI-S, the parental decision and other reasons, the RR was 1.69 (95% CI 1.21–2.36) and 1.70 (95% CI 1.07–2.69), respectively, p < 0.05, when using OLBI-S the RR was 1.76 (95% CI 1.28–2.42) and 2.20 (95% CI 1.48–3.28), respectively, p < 0.001. This trend was especially noted among students whose parents worked in the health care system: burnout among such students was more common when comparing the results of parental and own decisions (45.9% vs 38.7% according to CBI-S, and 54.1% vs 41.3% according to OLBI-S), p < 0.001. Burnout was significantly more common among students dissatisfied with their chosen profession (Tables 1, 2). Moreover, such a relationship was more significant among 1st and 2nd year students (p ≤ 0.001), 5th-year students (p < 0.05), and interns (p < 0.001).

74 (13.5%) of 548 respondents indicated that they smoke. Of these, 27 (36.5%) smoked before entering a medical university, while 47 (63.5%) started smoking after entering university. Burnout was more common among students who smoke. Burnout (based on OLBI-S) was found to be two times more common among students who started smoking after admission to medical school (RR = 1.98, p < 0.05). Among students who started smoking before entering university, 83% indicated that they began to smoke more during their studies; moreover, they were 7 times more likely (RR = 7.4, p < 0.05) to experience PB. Smoking habits were associated with all dimensions of CBI-S and OLBI-S with the exception of CRB (Table 2). Of the 547 students, 127 (23.2%) drink alcohol. Burnout (according the OLBI-S criteria) was 1.93 times more common among alcohol users (Table 1), this was especially due to the high level of disengagement (RR = 2.03, p < 0.001), but association with another burnout dimensions also was found (Table 2).

A relationship testing by χ2-test (Table 2) reveals that the prevalence of PB (p < 0.05) and high level disengagement (p < 0.001) was significantly higher in women and in students with an additional part-time job. Furthermore, students with extracurricular activities (participation in scientific clubs, student societies, and volunteering) comparison to students without such activities show higher prevalence of CRB (p < 0.05).

Lastly, we evaluated the relationship between burnout and various medico-social and psychosomatic consequences. For this, statistical methods were used, such as chi-square test, correlation and regression analysis. Respondents with positive answers for the following questions had a significant more expressed burnout for all dimensions of CBI-S and OLBI-S: suicidal ideation, thoughts of dropping out, interpersonal relationship problems with family/friends, dissatisfaction with academic performance and chosen profession. In addition, students indicated pursuits of high parental expectations were more prone to PB, SRB, TRB, high level of exhaustion and disengagement.

Associations of burnout with headaches, tiredness, sleep disturbances, depression, and anxiety on log-linear regression analysis presented in Table 3. To do this, students were divided into two groups of “burnout” and “non-burnout” according to the Christensen criterion for CBI-S [36] and the past validation study for OLBI-S [34]. We also note that the correlation analysis of burnout levels, both according to the CBI-S and OLBI-S scales, with the levels of psychosomatic indicators was significant (r = 0.313–0.624, p < 0.001). As presented in Table 3, having academic burnout was associated with various psychosomatic conditions. Moreover, we compared the levels of depression and anxiety in groups of students with and without burnout according to the CBI-S and OLBI-S criteria based on the t-test. According to the CBI-S criteria, the PHQ-9 and GAD-7 scores of students with burnout were significantly higher compare to non-burnout students: 25.6 ± 6.03 vs 16.8 ± 5.44 (t = 16.3), and 19.2 ± 5.75 vs 12.5 ± 4.69 (t = 13.9), respectively (p < 0.001). This was also confirmed using the OLBI-S: in PHQ-9 scale among burnout students’ M = 24.5 (SD = 6.83), among non-burnout M = 16.9 (SD = 5.37) (t = 13.9); in GAD-7 this values were 18.2 ± 6.07 and 12.6 ± 4.83 (t = 11.3), respectively (p < 0.001).

**Table 3:** Burnout associations with somatic symptoms, depression and anxiety (N = 736)

Variables	Burnout (CBI-S)			Burnout (OLBI-S)		
	β (95% CI)	R^2^	F, p	β (95% CI)	R^2^	F, p
Headache	0.674 (0.495–0.853)	0.100	54.7, < 0.001	0.534 (0.358–0.711)	0.065	35.5, < 0.001
Tiredness	1.010 (0.843–1.180)	0.219	142, < 0.001	0.959 (0.796–1.120)	0.210	134, < 0.001
Sleep disturbance	0.777 (0.601–0.953)	0.130	75.2, < 0.001	0.642 (0.468–0.816)	0.094	52.5, < 0.001
Depression (PHQ-9)	1.270 (1.120–1.420)	0.346	267, < 0.001	1.100 (0.944–1.260)	0.277	193, < 0.001
Anxiety (GAD-7)	1.130 (0.974–0.290)	0.277	193, < 0.001	0.937 (0.774–1.100)	0.201	127, < 0.001

*CBI-S* Copenhagen burnout inventory-students survey, *OLBI-S* Oldenburg burnout inventory for college students

## Discussion

The prevalence of burnout among medical students in Kazakhstan, identified using the CBI-S and OLBI-S, was 28% and 31%, respectively. In one systematic review of 24 studies of 17,431 pre-residency medical students, overall burnout prevalence across the entire student population was estimated to be 44.2% (33.4–55.0%) [4].

Our study revealed that while prevalence of burnout was not dependent on gender, women showed significantly higher rates of personal burnout (p < 0.05). Using the OLBI-S, we found that a higher level of disengagement was found among male students (Table 2).

### Burnout and decision to study medicine

In this work, it was found that when admission to a medical school is the decision of the student him/herself, or that he/she believes that it was, then during the study period, the student is likely to reported significantly lower levels of burnout (p < 0.001) than student counterparts who chose medical education for other reasons, including the urging of parents and close relatives. It has also been observed that students whose parents work in the health care system are more prone to burnout if the decision to admit medical school was chosen by the parents. Thus we determined that a dependent decision of the student to enter a medical school was a strong predictor for burnout development.

Burnout was also associated with high parental expectations. Thus, students who noted that they are pursued by high parental expectations are 1.7 times more likely to burnout, which is observed across all burnout dimensions, except for CRB. Moreover, students who were dissatisfied with their chosen profession were 2.4–3.2 times more likely to experience burnout (Tables 1, 2). When broken down into courses of study, significant differences were obtained among the following students: 1st, 2nd, 5th year undergraduate, and internship students. The obtained differences among students of the 1st and 2nd year of study, presumably, can be explained by the fact that this is from the 1st years in medical school and they are adapting, realizing their chosen profession. The 5th course on the Kazakhstani system of medical education is special in that this is the final year before receiving a bachelor's degree in medicine, and graduation courses again face the question: get a diploma and leave medicine or continue training in an internship to become a practitioner. At the same time, a significant relationship between burnout and satisfaction with the chosen profession among interns can be interpreted as the following potential reasons: firstly, during the internship, students receive more hours of practical training and have more contacts with patients; and secondly, earlier, students entering the internship had already chosen the direction (general practitioner (GP), therapy, surgery, obstetrics and gynecology, or pediatrics), but later, due to the development of primary health care in Kazakhstan, the internship began to be carried out only GP specialty, while deeper specialization began to receive only on residency.

### Burnout and academic life

According to Dyrbye et al. [43], medical students likely to suffer burnout as they advance in their medical training. In current study, burnout prevalence among medical students was also different according to the year of study; 2–5 undergraduate students, interns, and residents were more pronounce burnout compare to 1 year students (Table 1, Fig. 1). This was especially noticeable when using the OLBI-S.

In the current study, authors wanted to study the impact of extracurricular activities such as participating in scientific clubs, student societies and volunteering on burnout. We found that students with extracurricular activity had a lower rate of personal burnout and was less disengaged, but showed higher level of colleague-related burnout comparison to students without it (p < 0.05). We also found a relationship between place of residence and academic burnout. Regardless of the measurement method, burnout was less common among students living in the dormitory. Participation in extracurricular activities and living in a dormitory are united by the fact that students can maintain communication in a circle of like-minded people; although they may get tired of excessive such communication. Thus, it was noted that participants indicated the presence of relationship problems with family and friends were more prone to burnout. Strong relations between stress and interpersonal relationship problems among medical students were described earlier by Salam et al. [44], Bhagat et al. [45].

Burnout is known to be associated with serious thoughts of dropping out [46]. In the current study we found the same association: students who report thoughts of dropping out from medical school show significantly higher burnout prevalence (RR = 3.3 (CBI-S), 3.9 (OLBI-S), p < 0.001). Moreover, we found that students who are not satisfied with their academic performance have more pronounced burnout syndrome (p < 0.001). According to Dyrbye et al. [47], thoughts of dropping out can be described as the manifestation of distress.

### Burnout and part-time job

Many students during the training period take on additional work for various reasons: to make extra money or, try to improve their practical skills by working as junior medical personnel in clinics. Students doing additional work outside of the required curriculum represented 25% of our respondents. These students were found to have significantly higher prevalence of personal burnout (p < 0.05) and high level of disengagement (p < 0.001) compared to students who did not have any job; this was regardless of the place of work: in the medical or non-medical field. This can be explained by the presence of additional stress among students who combine study and work, and less time spent at the university, as a result of a decrease in satisfaction with academic life [48].

### Burnout and mental health

Our observations revealed significant positive associations of burnout with somatic symptoms (headache, fatigue, and sleep disturbances), anxiety, and depression (p < 0.001).

Our study found that students with suicidal ideation have more expressed burnout in total and across all studied dimensions (p < 0.05). Also, students who had suicidal ideation were almost 2.4–3.0 times more likely to develop burnout than those who did not have suicidal ideation (p < 0.001).

In the current research, 74 out of 548 students (13.5%) indicated that they smoke cigarettes, and smoker students had higher burnout prevalence (RR = 1.6–3.3, p < 0.05). Moreover, burnout was more common among students who started smoking after admission to medical school. Among students who started smoking before entering university, increased smoking was positively associated with personal burnout.

In this study, we found that higher burnout prevalence was found in students who drink alcohol (RR = 1.9, p < 0.001).

The resulting differences in the prevalence of burnout and the degree of association with certain factors when using CBI-S or OLBI-S can be explained by differences in the measured components of burnout. Thus, OLBI-S includes exhaustion and disengagement dimensions, while, CBI-S focuses only on exhaustion in different life domains (PB, SRB, CRB and TRB).

### Study limitations

Some limitations need to be considered in this study. First, this study is cross-sectional; further cohort studies are needed to determine more accurate results. Secondly, it must be taken into account that the training system in different courses is different. So junior students are trained in a linear model, while senior students are trained in a cyclical way. Therefore, it is impossible to predict the influence of additional factors of the academic environment on the level of burnout for correct comparison of results. Thirdly, the sample presented in this study is not random, since any students who received the mailing list could take the survey. It is assumed that students with high rates of academic burnout are less interested in participating in research, which may lead to an erroneously reduced burnout rate in the population. Moreover, the data were obtained among students only from one university, and generalization of the results to all Kazakhstani medical students is not acceptable.

## Conclusion

The prevalence of burnout syndrome among Kazakhstani medical students is quite high. A significant relationship of burnout with a student’s gender, year of study, accommodation, academic performance, extracurricular activities, and social, personal, and psychosomatic state of students was revealed. Longitudinal studies are required to further explore and elucidate the patterns of burnout among medical students. The data obtained can help in organizing psychological assistance for medical students in Kazakhstan.

## Data Availability

The datasets used and/or analysed during the current study available from the corresponding author on reasonable request.
